# Radiation-induced myocardial metabolic impairment detected by ¹²³I-BMIPP/⁹⁹ᵐTc-MIBI metabolic–perfusion mismatch following thoracic radiotherapy for thymoma: a case report

**DOI:** 10.1007/s13691-026-00868-0

**Published:** 2026-04-22

**Authors:** Masaki Watanabe, Yaichiro Hashimoto, Maiko Sone, Kyoko Oguri, Sakura Nishiwaki, Michinobu Nagao, Shuji Sakai

**Affiliations:** 1 Department of Diagnostic Imaging and Nuclear Medicine, Tokyo Women’s Medical University, Tokyo, Japan; 2 Department of Radiation Oncology, Tokyo Women’s Medical University, 8-1, Kawada-cho, Shinjuku-ku, Tokyo, 162-8666 Japan; 3 Department of Cardiology, Tokyo Women’s Medical University, Tokyo, Japan

**Keywords:** Radiation-induced heart disease, Myocardial metabolic impairment, ¹²³I-BMIPP, ⁹⁹ᵐTc-MIBI, Metabolic–perfusion mismatch, Thoracic radiotherapy

## Abstract

Radiation-induced heart disease is a well-recognized late complication of thoracic radiotherapy and comprises a broad spectrum of cardiac disorders. Because myocardial metabolic impairment may precede overt structural or perfusion abnormalities, early or subclinical myocardial injury is often challenging to detect using conventional cardiac imaging modalities. We report a case of late-onset radiation-induced myocardial metabolic impairment identified by dual myocardial scintigraphy many years after thoracic radiotherapy for thymoma. The patient, a man diagnosed with malignant thymoma at 42 years of age, underwent two courses of thoracic radiotherapy during the course of his illness. The first course was confined to the mediastinum without cardiac exposure, whereas the second course, delivered at 52 years of age for recurrent disease with pericardial and myocardial invasion, resulted in direct cardiac irradiation. After a prolonged asymptomatic period, at 67 years of age (approximately 15 years after the second course of radiotherapy), the patient developed symptoms consistent with heart failure. Coronary angiography revealed no significant coronary artery stenosis. Dual myocardial scintigraphy demonstrated a characteristic metabolic–perfusion mismatch: reduced uptake of ¹²³I-β-methyl-p-iodophenyl-pentadecanoic acid (BMIPP) in the anterior to lateral wall of the left ventricle, precisely corresponding to the irradiated cardiac region, while myocardial perfusion assessed by ⁹⁹ᵐTc-methoxyisobutylisonitrile (MIBI) was preserved. Retrospective dosimetric analysis demonstrated a mean heart dose of 12.8 Gy during the second course of radiotherapy, supporting a radiation-induced mechanism of myocardial impairment. This case highlights the clinical value of combined ¹²³I-BMIPP and ⁹⁹ᵐTc-MIBI myocardial scintigraphy for the noninvasive detection of late-onset radiation-induced myocardial impairment in long-term cancer survivors, even in the absence of obstructive coronary artery disease.

## Introduction

Radiation therapy is an essential component of curative and adjuvant management for various thoracic malignancies, including esophageal cancer, breast cancer, malignant lymphoma, and thymoma. As cancer survival continues to improve, greater emphasis has been placed on the late adverse effects of treatment, particularly radiation-induced heart disease (RIHD), which can substantially contribute to long-term morbidity and mortality in cancer survivors [1–3].

RIHD comprises a broad spectrum of cardiac complications, including coronary artery disease, pericardial disease, valvular dysfunction, conduction abnormalities, and cardiomyopathy. These conditions typically manifest after a long latency period, often spanning several years to decades following irradiation, thereby making early diagnosis challenging. Moreover, conventional diagnostic tools such as echocardiography, electrocardiography, and coronary angiography may fail to detect subtle or early myocardial impairment, particularly in patients without obstructive coronary artery disease [1, 2].

Previous studies have established a dose–response relationship between cardiac radiation exposure and the subsequent risk of RIHD, with mean heart dose identified as a clinically significant dosimetric parameter. Darby et al. reported that the risk of major coronary events increases linearly with rising mean heart dose, even at relatively low dose levels, underscoring the absence of a safe threshold for radiation-related cardiac injury [3]. Although these findings primarily pertain to ischemic cardiac events, they highlight the importance of cardiac radiation exposure in the development of late-onset cardiac toxicity.

Nuclear cardiology provides a unique, noninvasive approach to evaluating myocardial impairment by enabling separate assessment of myocardial metabolism and perfusion. ¹²³I-β-methyl-p-iodophenyl-pentadecanoic acid (BMIPP) reflects myocardial fatty acid metabolism and is particularly sensitive to mitochondrial dysfunction, whereas ⁹⁹ᵐTc-methoxyisobutylisonitrile (MIBI) evaluates myocardial perfusion. A discrepancy between these two imaging modalities, referred to as metabolic–perfusion mismatch, may indicate myocardial impairment in which metabolic abnormalities precede overt perfusion defects [4–6]. In addition, focal myocardial uptake on ¹⁸F-FDG PET has been reported within the irradiated field after chemoradiotherapy for esophageal cancer [7]. Nevertheless, reports describing this imaging pattern in the context of late-onset RIHD remain scarce [8, 9]. Previous reports and studies of functional nuclear imaging for RIHD after thoracic/mediastinal radiotherapy are summarized in Table 1.

**Table 1:** Summary of published studies using functional nuclear imaging to detect radiation-induced myocardial injury after thoracic/mediastinal radiotherapy

First author (ref)	Year	Population/setting	Imaging modality	Timing from RT	Key findings relevant to radiation-induced myocardial injury
Umezawa [4]	2013	Esophageal cancer; complete response > 6 months after curative RT (*n* = 34)	¹²³I-BMIPP scintigraphy with CT-based 15-segment model	> 6 months (median 22 months; range 6–103)	Reduced BMIPP uptake was observed within irradiated myocardium; segments receiving ≥ 40 Gy showed significantly greater metabolic impairment, suggesting a dose-dependent effect.
Umezawa [5]	2015	Esophageal cancer; mediastinal RT (prospective pilot, *n* = 5)	¹²³I-BMIPP SPECT/CT	Baseline and 6 months after RT	BMIPP defect score increased after RT; defects corresponded to the myocardial dose distribution, indicating subclinical metabolic impairment detectable at 6 months.
Takanami [6]	2016	Esophageal cancer; definitive chemoradiotherapy (prospective, *n* = 12)	¹²³I-BMIPP SPECT/CT	Pre-CRT, pre-boost, 3 months and 1 year after CRT	Heart dose parameters (e.g., mean heart dose/Vx) correlated with worsening BMIPP uptake; metabolic impairment was detectable early and could persist at 1 year.
Jingu [7]	2006	Thoracic esophageal cancer after chemoradiotherapy (*n* = 64)	¹⁸F-FDG PET	≥ 3 months after CRT	Focal increased FDG uptake in basal myocardium within irradiated fields was observed in 20.3%; in examined cases, corresponding low BMIPP and/or ²⁰¹TlCl uptake and MRI abnormalities suggested radiation-induced myocardial damage.

Abbreviations: BMIPP, β-methyl-*p*-iodophenyl-pentadecanoic acid; CRT, chemoradiotherapy; FDG, fluorodeoxyglucose; LV, left ventricle; RT, radiotherapy; SPECT/CT, single-photon emission computed tomography/computed tomography

## Case report

A 42-year-old man initially presented with eyelid ptosis and was subsequently diagnosed with myasthenia gravis based on a positive edrophonium test. Chest computed tomography revealed an anterior mediastinal mass consistent with thymoma.

The patient underwent extended thymectomy via median sternotomy for the treatment of myasthenia gravis and thymoma. Complete resection was not achievable because the tumor was firmly adherent to the aorta; therefore, partial tumor biopsy with lymph node sampling was performed. Histopathological examination confirmed an invasive thymoma, Masaoka stage III, without lymph node metastasis. Postoperatively, residual disease was treated with thoracic radiotherapy to the anterior mediastinum, using 10-MV X-rays with anterior oblique opposed fields, to a total dose of 60 Gy in 30 fractions, in combination with two cycles of chemotherapy consisting of cyclophosphamide, vincristine, and prednisolone. This initial course of radiotherapy was limited to the mediastinum and did not involve cardiac exposure. Following the initial course of radiotherapy, the patient developed radiation pneumonitis, which improved with intensification of corticosteroid therapy.

Several years later, long-term corticosteroid therapy for myasthenia gravis resulted in osteonecrosis of the femoral head, necessitating left total hip arthroplasty. Subsequently, worsening myasthenic symptoms required hospitalization, and additional immunosuppressive therapy with tacrolimus was initiated alongside prednisolone and pyridostigmine.

**Fig. 1:**
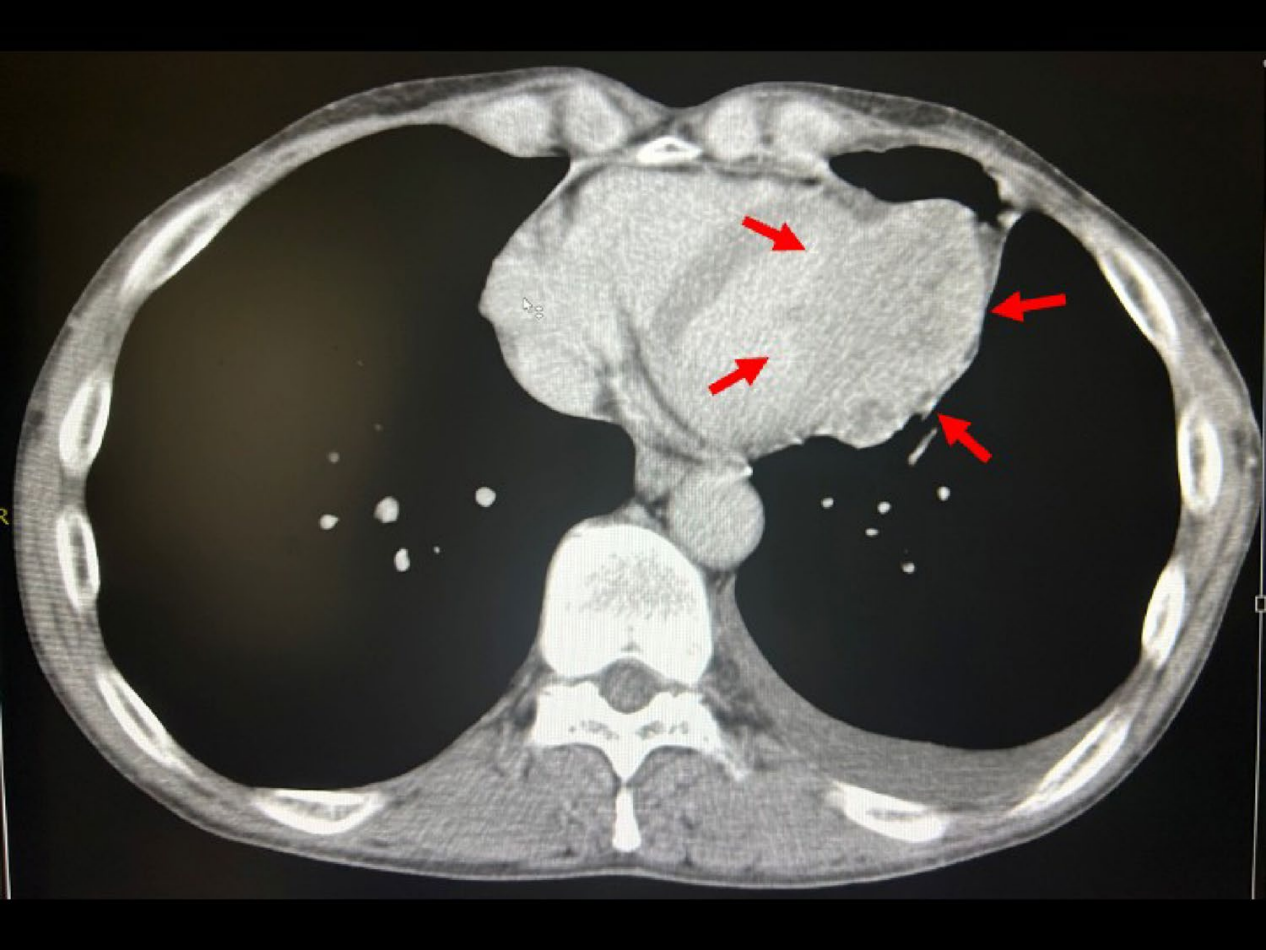
Contrast-enhanced CT of recurrent thymoma with myocardial invasion. Contrast-enhanced computed tomography shows a recurrent thymoma infiltrating the lateral wall of the left ventricle with pericardial involvement. A well-enhancing mass measuring approximately 60 mm is observed

**Fig. 2:**
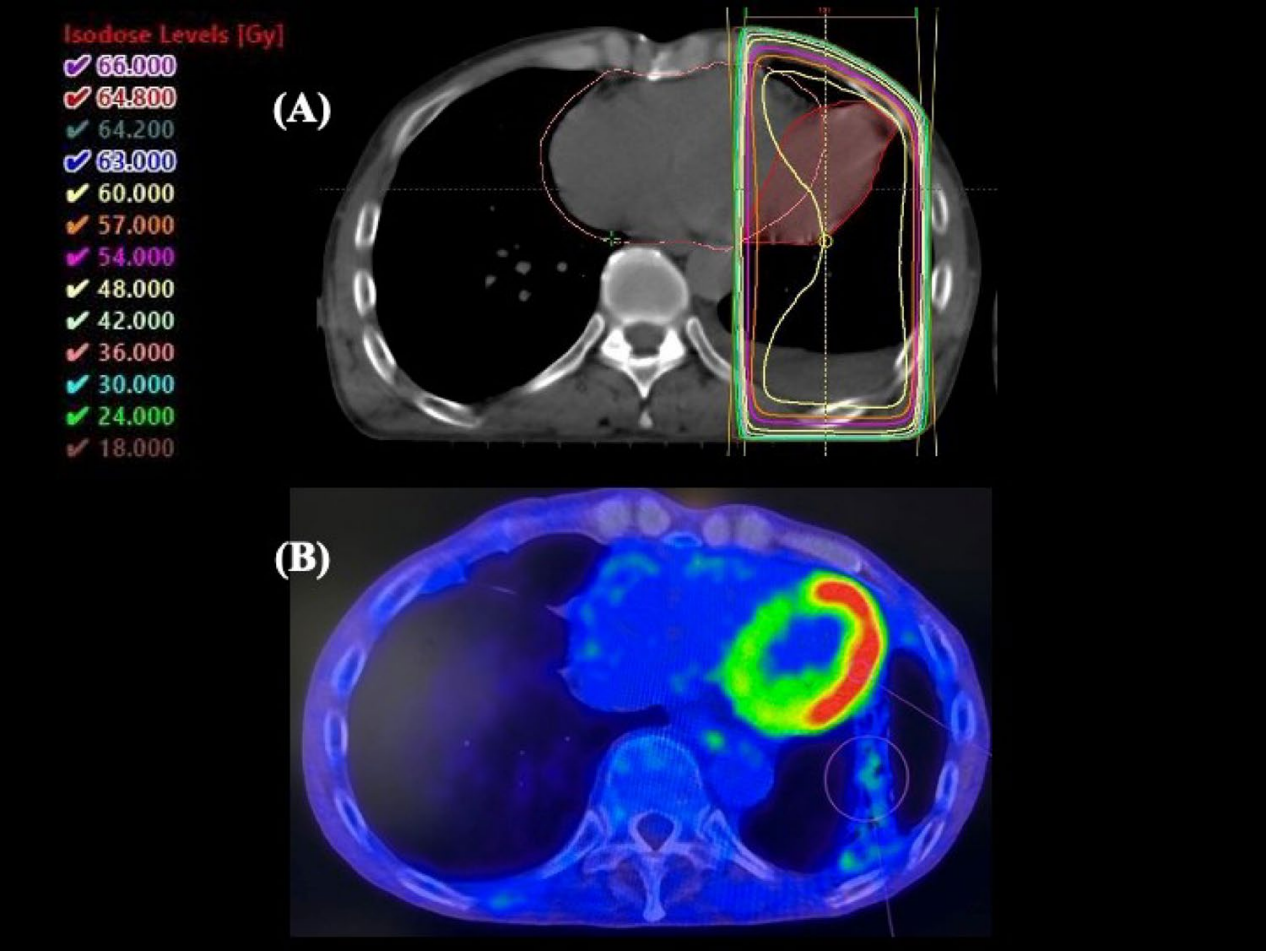
Spatial correlation between radiation dose distribution and myocardial metabolic abnormality. **(a**) Dose distribution of the second course of thoracic radiotherapy, demonstrating the high-dose region involving the anterior to lateral wall of the left ventricle. (**b**) ¹⁸F-fluorodeoxyglucose positron emission tomography/computed tomography revealing focal increased glucose uptake in the corresponding myocardial region

Later in the disease course, the patient developed progressive dyspnea, generalized fatigue, and fever. Imaging studies revealed pleural and pericardial effusions, and contrast-enhanced computed tomography demonstrated a 60-mm enhancing mass infiltrating the lateral wall of the left ventricle with pericardial involvement, findings consistent with recurrent malignant thymoma (Fig. 1). Pericardial and pleural drainage were performed, followed by pleurodesis. Subsequent video-assisted thoracoscopic biopsy and pericardial fenestration confirmed recurrent thymoma with pericardial and myocardial invasion. At the age of 52, salvage thoracic radiotherapy was administered to the recurrent lesion using 10-MV X-rays with an anteroposterior–posteroanterior opposing-field technique, delivering a total dose of 60 Gy in 30 fractions (Fig. 2a), which resulted in complete radiologic remission. In contrast to the initial treatment course, this second course of radiotherapy involved direct cardiac exposure due to pericardial and myocardial invasion.

The patient remained clinically stable for an extended period thereafter. At a follow-up visit, he was evaluated by the cardiology department for palpitations; however, no significant cardiac abnormalities were identified, and he was managed with observation alone.

After a long asymptomatic period, at the age of 67, approximately 15 years after the second course of thoracic radiotherapy, the patient developed exertional dyspnea and anorexia. He was initially treated at another institution for presumed pneumonia; however, his symptoms progressively worsened, and bilateral lower-extremity edema subsequently developed. Positron emission tomography demonstrated no evidence of recurrent thymoma, and he was admitted to our institution for further evaluation.

The patient had no history of diabetes mellitus, chronic kidney disease, hypertension, or dyslipidemia. He had a smoking history of approximately 20 cigarettes per day from 16 to 53 years of age.

Cardiac magnetic resonance imaging was not performed. Transthoracic echocardiography revealed right atrial enlargement and inferior vena cava dilation, with reduced left ventricular systolic function (left ventricular ejection fraction, 41%). Moderate mitral regurgitation with mitral valve prolapse was also noted. Laboratory investigations revealed an elevated B-type natriuretic peptide level. To exclude ischemic heart disease, coronary angiography was performed, which revealed no significant stenosis in the major coronary arteries.

**Fig. 3:**
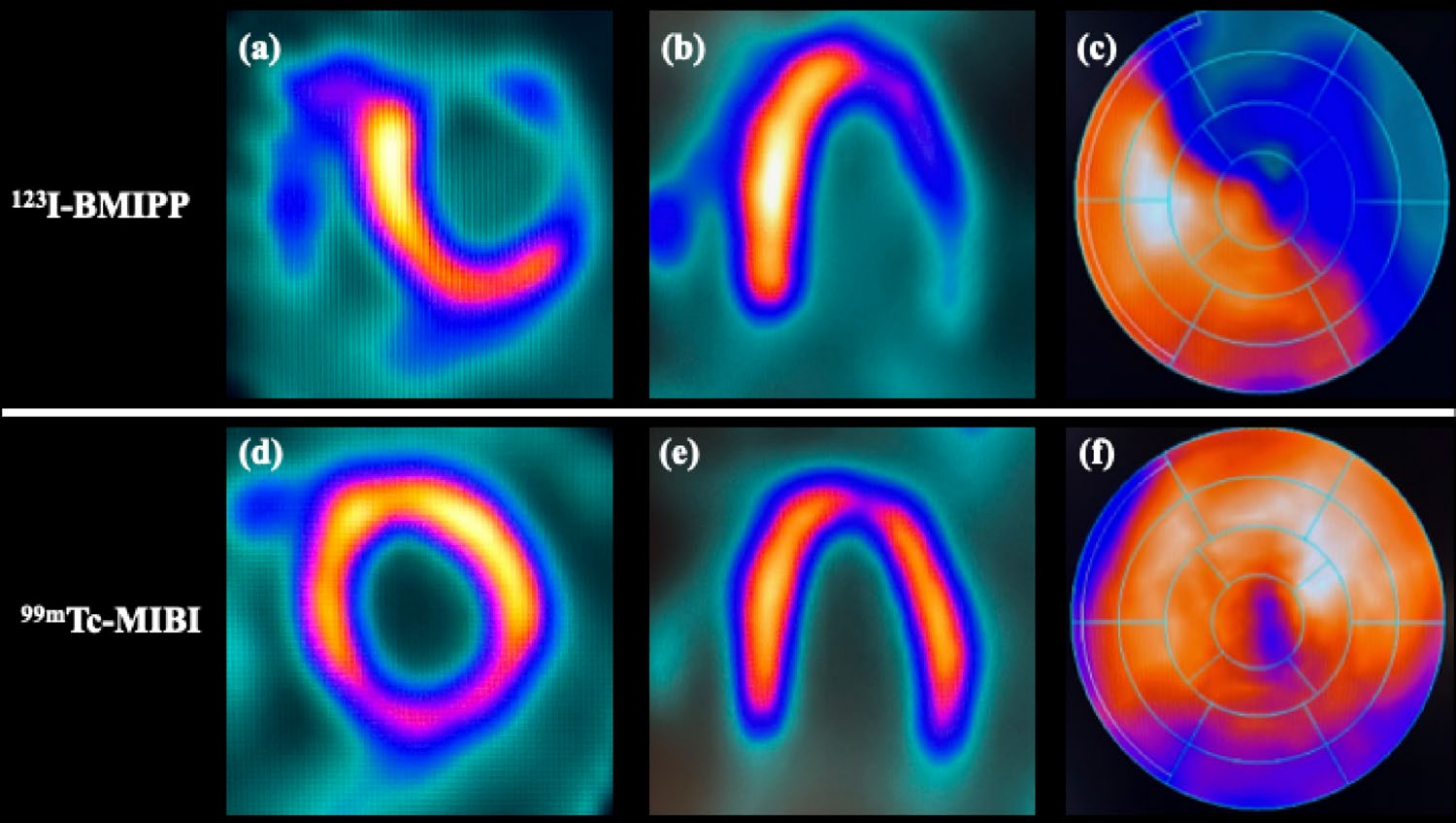
Dual myocardial scintigraphy demonstrating metabolic–perfusion mismatch. (**a**) ¹²³I-β-methyl-*p*-iodophenyl-pentadecanoic acid (BMIPP) single-photon emission computed tomography (SPECT) in the axial view demonstrates a well-demarcated reduction in tracer uptake in the anterior to lateral wall of the left ventricle. (**b**) ¹²³I-BMIPP SPECT in the coronal view shows concordant reduction in tracer uptake in the anterior to lateral left ventricular wall. (**c**) ¹²³I-BMIPP SPECT polar map (bull’s-eye plot) demonstrates a regional reduction in tracer uptake in the corresponding anterior to lateral left ventricular segments. (**d**) Corresponding axial ⁹⁹ᵐTc-methoxyisobutylisonitrile (MIBI) SPECT demonstrates preserved myocardial perfusion in the same region. (**e**) Corresponding coronal ⁹⁹ᵐTc-MIBI SPECT demonstrates preserved perfusion in the corresponding region. (**f**) ⁹⁹ᵐTc-MIBI SPECT polar map (bull’s-eye plot) demonstrates preserved perfusion in the same segments, facilitating objective visualization of the metabolic–perfusion mismatch and its regional extent. Note: As computed tomography-based attenuation correction was not available, mild inferior–posterior reduction on ⁹⁹ᵐTc-MIBI may reflect attenuation

Subsequent myocardial scintigraphy was conducted to assess myocardial metabolism and perfusion. Dual-isotope ⁹⁹ᵐTc/¹²³I imaging was acquired using a dedicated cardiac cadmium-zinc-telluride single-photon emission computed tomography system without computed tomography, precluding computed tomography-based attenuation correction. BMIPP single-photon emission computed tomography showed a well-demarcated reduction in tracer uptake in the anterior to lateral wall of the left ventricle (Fig. 3a and c), whereas MIBI scintigraphy demonstrated preserved perfusion in the corresponding region (Fig. 3d and f). The area of metabolic–perfusion mismatch precisely corresponded to the previous high-dose radiation field involving the heart. Fluorodeoxyglucose positron emission tomography, performed to evaluate for thymoma recurrence, demonstrated no abnormal uptake indicative of recurrent malignancy. However, focal increased FDG uptake was noted in the anterior to lateral wall of the left ventricle (Fig. 2b). Retrospective dose–volume analysis revealed a mean heart dose of 12.8 Gy during the second course of radiotherapy, with the anterior left ventricular wall receiving a maximum dose of 61.7 Gy.

Based on these imaging findings and the long-term clinical course, a diagnosis of non-ischemic heart failure secondary to radiation-induced myocardial metabolic impairment was established. Standard medical therapy for heart failure resulted in symptomatic improvement, and the patient was discharged with planned outpatient follow-up.

## Discussion

This case demonstrates late-onset radiation-induced myocardial metabolic impairment, evidenced by a characteristic metabolic–perfusion mismatch on dual myocardial scintigraphy approximately 15 years after thoracic radiotherapy with direct cardiac exposure. Importantly, the patient developed symptomatic heart failure after a long latency (> 15 years), highlighting the need for long-term surveillance in thoracic radiotherapy survivors. The absence of obstructive coronary artery disease, together with the precise spatial correspondence between the myocardial metabolic abnormality and the irradiated cardiac region, strongly supports radiation-induced myocardial injury as the underlying mechanism of heart failure in this patient [1, 2].

Radiation-induced myocardial injury is a multifactorial process involving microvascular damage, endothelial dysfunction, mitochondrial impairment, and progressive myocardial fibrosis [1, 2]. Mitochondrial dysfunction leads to impaired fatty acid utilization and reduced adenosine triphosphate production, which may precede detectable structural abnormalities or perfusion impairment. Because myocardial fatty acid metabolism is highly dependent on intact mitochondrial oxidative capacity, metabolic abnormalities can be identified earlier than perfusion defects. In addition, radiation-associated endothelial injury and microvascular dysfunction may contribute to metabolic impairment even when perfusion appears preserved on conventional perfusion single-photon emission computed tomography because microvascular abnormalities do not necessarily manifest as overt perfusion defects.

BMIPP reflects myocardial fatty acid metabolism, whereas MIBI primarily reflects myocardial perfusion. A metabolic–perfusion mismatch pattern, characterized by reduced BMIPP uptake with preserved MIBI perfusion, has been reported as an indicator of myocardial injury in which metabolic dysfunction precedes overt perfusion abnormalities [4–6]. In the present case, this mismatch most likely indicates chronic, localized metabolic impairment of the irradiated myocardium rather than acute ischemia, a finding consistent with the long latency following cardiac irradiation [4, 5]. This interpretation is further supported by retrospective dosimetric analysis, which demonstrated that the myocardial segment with reduced BMIPP uptake corresponded to an area exposed to a relatively high radiation dose [4–6]. In the present case, mild inferior–posterior reduction on the 99mTc-MIBI images may partly reflect attenuation or relative normalization differences between tracers, as computed tomography-based attenuation correction was not available. Although the patient previously received cyclophosphamide, a potentially cardiotoxic agent that may reduce cardiac reserve as a “second hit,” the focal distribution of the metabolic abnormality corresponding to the irradiation field supports radiation-induced injury as the predominant mechanism in this case.

Previous studies have established a clear dose–response relationship between cardiac radiation exposure and the subsequent risk of RIHD, with mean heart dose recognized as a clinically relevant dosimetric parameter. Darby et al. reported that the risk of major coronary events increases linearly with increasing mean heart dose, even at relatively low dose levels, indicating the absence of a safe threshold for radiation-related cardiac injury [3]. Subsequent reviews and expert consensus statements have emphasized the importance of minimizing cardiac dose during thoracic radiotherapy to reduce long-term cardiac morbidity [1, 2, 10].

In the present case, retrospective dose–volume analysis revealed a mean heart dose of 12.8 Gy during the second course of radiotherapy, with the anterior to lateral left ventricular wall receiving a maximum dose of 61.7 Gy. Although a causal relationship cannot be inferred from a single case, these dosimetric findings align with existing evidence linking cardiac radiation dose to late-onset cardiac injury. Notably, the myocardial region exhibiting reduced BMIPP uptake precisely corresponded with the region receiving the highest radiation dose, indicating that both mean heart dose and regional dose heterogeneity may contribute to chronic radiation-induced myocardial metabolic impairment. Furthermore, focal fluorodeoxyglucose uptake was noted in the irradiated myocardial region, indicating a metabolic shift from fatty acid to glucose utilization, a pattern consistent with chronic myocardial injury and further supporting the diagnosis of radiation-induced myocardial damage. Alternatively, focal FDG uptake in the chronic phase could also indicate ongoing tissue remodeling and/or low-grade inflammation within the irradiated myocardium.

This case underscores the importance of considering RIHD in patients presenting with unexplained heart failure long after thoracic radiotherapy, even when obstructive coronary artery disease or perfusion defects are absent [1, 2, 10]. A history of direct cardiac exposure during radiotherapy, rather than cumulative mediastinal irradiation alone, should prompt careful evaluation for late-onset myocardial injury. Assessing both myocardial metabolism and perfusion using ¹²³I-BMIPP and ⁹⁹ᵐTc-MIBI provides complementary diagnostic insights, enabling noninvasive detection of chronic radiation-induced myocardial damage [4–6, 11]. As the population of long-term cancer survivors continues to grow, increased awareness of late cardiac toxicity and appropriate use of nuclear cardiology imaging will be essential for timely diagnosis and optimal management [10, 11].

## Data Availability

Data sharing is not applicable to this study, as no datasets were generated or analyzed.
